# Clinic of Professor Dunglison

**Published:** 1845-04

**Authors:** Samuel G. White

**Affiliations:** Georgia


### CLINICAL LECTURES AND REPORTS.

### PHILADELPHIA HOSPITAL.

*Saturday, January 4, 1845.*

### CLINIC OF PROFESSOR DUNGLISON.

Reported by Dr. Samuel G. White, of Georgia.

Professor Dunglison considered it unnecessary to introduce the patients who were the subjects of remark at the previous clinic, as, from their chronicity, long attendance would be required to observe any effect from the treatment prescribed.

The female, with enlargement of the cervical glands and inflammation of the scalp, had been sent out of the hospital to enjoy the benefit of fresh air, and has already returned much improved. She will continue the use of the ferri iodidum, which had been prescribed with the intention of invigorating the constitution, and increasing the plasticity of the blood. These cases may form the subject of some future report.

The professor then proceeded to investigate

DISORDERS OF THE BILIARY SECRETION, the physiology of the liver having been under consideration during the week at the Jefferson Medical College.

Disorders of this secretion are not of unfrequent occurrence in certain seasons, whilst they are rare in others. During the whole of the clinical course of last year, the lecturer was unable to exhibit to the class a single case of hepatic affection,—primary or secondary. At one time, almost every gastric or intestinal affection was considered to be caused by some modification in the secretion of the liver, or to be—as it was termed—"bilious;" and even members of the profession have treated themselves for years for hepatic disease, and yet the liver on dissection has been found unaffected. The lecturer alluded to a striking instance of this kind in an eminent London hospital physician.

Hepatic affections are not, however, so frequent at present as they were some years since, which is to be chiefly ascribed to the temperance reformation. Their most common cause is unquestionably the free use of ardent spirits, the modus operandi being sufficiently obvious. Whenever fluids of a certain tenuity are taken into the stomach, they readily pass by endosmose into the portal vessels, and are thence conveyed to the liver. Should large quantities of alcoholic drink be thus received and circulated through the liver, it can easily be comprehended, that the excessive stimulation thus induced may modify the nutrition of that viscus, and, as a consequence, organic disease or functional derangement may supervene. Hence we can comprehend why inebriates should be most liable to this class of diseases.

The term "bilious," as ordinarily used, denotes a great variety of affections, often of the most opposite character, and it is frequently very difficult to determine the pathology of the affection which it is intended to designate. In some portions of the country, practitioners are in the habit of applying it to states of the system wholly different—for example, to states in which there may be an augmentation, a diminution or a vitiation of the biliary secretion.

The lecturer considers organic primary disease of the liver to be by no means of frequent occurrence,—the so-called bilious diseases being, in truth, nothing more than functional derangements, consequent on irritation seated in the alimentary canal. Irritation, or inflammation of the gastro-enteric mucous membrane, is propagated along its extension, constituting the lining of the ductus communis choledochus, to the liver, producing, as in other cases—if the inflammation be violent—first, an arrestation, and, afterwards, an augmentation of the secretion of bile, which in the latter case is generally morbid; and the individual is in both cases said to be "bilious."

Structural disease of the liver was never perhaps so common as was formerly supposed, whilst functional aberrations, the consequence of irritation of the gastro-intestinal mucous membrane, are not unfrequent.

The professor here introduced two patients labouring under

JAUNDICE, neither—in all probability—the result of serious organic disease, but of functional disturbance of the liver.

The term jaundice—from the French *jaunisse*—is employed to denote an affection, characterized by yellowness of the surface and conjunctiva. From the colour alone it may generally be diagnosticated; but in hospitals, and more especially in military hospitals, caution is at times requisite in pronouncing on the nature of the affection. They, who are desirous of evading duty—well known as "malingerers"—may feign jaundice by tinging the surface with turmeric or some other yellow colouring matter, and thus deceive the inexperienced. The fraud may, however, be always detected by examining the conjunctiva, which cannot be tinged by any artificial means, whereas, in true jaundice it is always more or less yellow. Whenever, therefore, this tunic retains its natural hue, a suspicion of the true nature of the case may be indulged.

Considerable discrepancy has existed among writers as to the cause of the yellowness of the surface in jaundice. The professor thinks it is in every case attributable to the presence of bile in the blood. Some, however, who believe that no foreign material can be absorbed into the blood unchanged, without injurious consequences to the economy, ascribe it to a disease of the red corpuscles of the blood; but that the serum alone is tinged, and that the hue is owing to the presence of bile, seems proved by the greenish colour which jaundiced serum assumes on the addition of nitric acid. Moreover, when the red corpuscles are examined by the microscope, they appear to be unchanged.

The consideration of the phenomena of jaundice would seem to confirm this view of its pathology. All the tissues are tinged yellow, which is easily explicable, if we suppose the bile to be circulating through them. The secretions are also altered in colour, and especially the urine, which, on the addition of nitric acid, passes through a variety of shades, from mahogany to green, blue, and almost black. This fact, and the tinge communicated to the linen, seem to show, that the colouring matter of the bile is contained in the urine. On the subsidence of the jaundice, it assumes its natural colour. Moreover, the globules, if diseased, could not produce the colour, as they are not contained in the renal secretion. Again, in many cases of jaundice, some obstruction is found to exist, which prevents the ready passage of the bile into the intestines, as indicated by the white or clay-coloured stools. Should there be such obstruction, it is not difficult to understand, that the bile might pass by exosmose through the coats of the ducts, and by endosmose enter the blood vessels, and give rise to jaundice.

Not only, however, may the bile, after being secreted, be reabsorbed by the blood vessels:—from a diseased condition of the liver, which prevents its secretion, the elements may come together at the point at which they should have been separated; and, instead of being eliminated, may remain in the fluid of the circulation, as, under certain conditions, urea or kiesteine collects in the blood; and in such case jaundice may result.

The symptoms are usually well marked. At first there is loss of appetite, with pain in the hypochondrium or epigastrium—or both—uneasiness in the head—lassitude, and more or less depression of spirits. These symptoms continue for a longer or shorter period, when the eyes and skin are observed to be yellow.

The urine is yellow, or of a mahogany colour, tinging the linen with which it may come in contact. The bowels are generally constipated from a want of the usual secretions, and the fæces are whitish or clay-coloured. Pressure will frequently cause pain of the epigastrium, and if the liver be inflamed or diseased, there will be pain in the right hypochondrium—augmented on pressure. There is not unfrequently, also, delirium or disturbance of the cerebral functions, and even coma, the consequence of the circulation of bile through the encephalic neurine.

Coma is a most unfavourable symptom, and is regarded by Dr. Stokes as prognostic of a fatal termination. He states, indeed, that he never knew a case where it was well marked which recovered. The experience of the lecturer is not exactly in accordance with that of Dr. Stokes. Although he considers coma a very grave symptom, it is not necessarily fatal. Much difficulty exists in explaining the immediate cause of this coma, as nothing satisfactory is found on a post-mortem examination.

The causes of jaundice are numerous. Among the most frequent may be mentioned inflammation, or irritation of the gastro-enteric mucous membrane, which is extended by continuous sympathy to the liver. Obstructions of any kind to the passage of the bile along the ducts to the intestine, must also be a common cause; it is easy to understand, that if a gall-stone should form in the gall-bladder, and subsequently pass into the duct and obstruct it, absorption of the bile might ensue, and give rise to the disease. The same would be the effect of biliary concretions forming in the hepatic ducts, or in the ductus communis choledochus.

The lecturer thought it advisable here to direct the attention of the class to the painful affection caused by the PASSAGE of a GALL-STONE—the suffering from which is excruciating, and can be likened to nothing but that produced by the progress of a calculus along the ureter.

The diagnosis of this affection is readily made. The patient is usually found writhing in agony on the floor, with his pulse unexcited, and with violent pain in the epigastrium or right hypochondrium, which is augmented in paroxysms. This has been called by Dr. Mason Good, *chololithus meaus*, in contradistinction to *chololithus quiescens*, in which the calculus is stationary in the gall bladder, and gives no pain.

After the symptoms indicating "hepatic colic" have continued for a longer or shorter time, they may cease, and after a varying period recur. The cause of this cessation appears at times to be owing to the entrance of the concretion into the ductus communis choledochus; and the renewal of the pain to its passage through the extremity of that duct, which is smaller than the other portions. Having cleared the duct, it enters the duodenum; the patient is now free from any further pain, unless there be other concretions passing. If the alvine evacuations be examined, the gall-stone may be detected; yet we may fail in so doing, although the symptoms may have been unequivocal.

Sometimes great numbers collect in the gall-bladder, more than a thousand having been found in one case on dissection. The lecturer referred to the case of a medical gentleman, who informed him, that his gall-bladder was prominent below the ribs, owing to its containing calculi, which he could cause to rattle by pressing over the region. The gall-bladder, when thus distended, has been mistaken for an abscess, and been punctured. The presence of gall-stones does not necessarily give rise to jaundice, although the latter is often an accompaniment of the former.

One of the patients before the class has not been very intemperate. The jaundice is of nine month's continuance in him, but it is now yielding. In the commencement of the attack there were symptoms of gastro-enteric irritation. He never experienced much, if any, inconvenience from lying on the left side; and although the liver appears to be somewhat enlarged, it is not probably seriously diseased. In many cases of jaundice, the general health is so little affected, that the patient is enabled to pursue his ordinary vocations without much inconvenience.

The lecturer here exhibited a portion of urine from each patient, and showed the tinge communicated to linen immersed in it, which was yellow in both cases, but more markedly so in one than in the other. When nitric acid was added, the green hue before alluded to was produced, showing the presence of the bile. *It* was also less marked in one than in the other case, which at one time was quite severe, but is now convalescent. The green colour thus produced may gradually pass to blue, and then to red, but sometimes it becomes red immediately, without passing through the blue stage.

In some cases of jaundice, the peculiar phenomenon of yellow vision is present. It is rare, however, and Frank states it to occur in only five cases in a thousand. In the instances before the class it was absent; and the lecturer remarked that he never had seen a case of it. The cause of yellow vision has occasioned considerable dispute. We can conceive, that should the humors of the eye become tinged, it might occur,—but it may be a nervous affection, as it is sometimes seen in adynamic fever.

The treatment of gall stone will depend on the period of the disease. During its passage, antispasmodics, so called, are manifestly indicated,—not articles, usually classed as such, but those agencies that relax the system, as blood letting, the warm bath, &c. To allay spasm and pain, opium should be given in full doses, and repeated until the desired effect is produced. It is astonishing how much opium may be administered in these and other spasmodic affections, without inducing narcosis.

Conjoined with these means, others may be adopted that will favor the progression of the gall stones, as emetics, cathartics, &c. By the efforts of vomiting, the peristaltic action of the intestine is inverted, and a degree of traction is exerted on the biliary passages that may favor the transit of the stone. Cathartics, also, by accelerating the peristole, will have a similar effect, though to a less extent.

Should great tenderness exist over the epigastrium, venesection may be advantageously resorted to, which, as before stated, may also exert a relaxing influence on the spasm.

Should the patient be debilitated, or of broken down constitution we ought to pause before resorting to emetics, as there may be risk of rupturing the ducts.

In such cases it is safer to have recourse to purgation; but this also must not be carried too far. If there exist no contraindication, a mercurial cathartic, which acts upon the upper portion of the intestinal canal, may be given, followed by a saline cathartic.

When the liver is seriously diseased, articles that stimulate it to augmented secretion should not be given too freely, for manifest reasons.

Where jaundice is not complicated or connected with the passage of biliary calculi, the treatment will of course be somewhat different from that just detailed.

The majority of cases of simple jaundice, demand no treatment, and will gradually pass away of themselves. It is the usual practice, however, to give mercurials, to excite the liver, if it be torpid; purgatives to obviate constipation, and under the views mentioned above, tonics to support the energies of the constitution. This plan was pursued in one of the patients before the class,—who came in with much disturbance of the cerebral functions; great irregularity and turmoil in the action of the heart; debility, and other unfavorable symptoms;—and under it he has much improved.

In all cases, the presence of bile in the blood is a mere epiphænomenon, and therefore demands no special attention. The treatment should generally be temporizing. The cause should, of course, be always inquired into, and if possible removed. The simple fact, however, of a person having jaundice, is not a sufficient indication that mercurials, or any other special remedies, are required. If disease of the liver exist, it may not be improper or injudicious to prescribe small doses of calomel or blue pill to produce a new action, but it should only be carried so far as to affect the mouth slightly. In the greater number of instances, there is no manifest pathological condition to be treated, aud if nothing be given, the jaundice will usually disappear in course of time.

If this be the condition in most cases of jaundice, how absurd the remedies that have been employed, and not long since, to remove obstructions which were fancied to exist in the ducts in every case ; when ether and turpentine were given in the form of the Remède de Durande, still recommended by some, with the intention of dissolving gall-stones? The cases often recovered, but the result was in no wise effected by the remedy, but by the recuperative powers. How could it be expected that thirty drops of turpentine and ether should have any action on a stone in the hepatic duct? How could they pass in a state of concentration through the stomach and duodenum into the duct; come in contact with the obstruction and dissolve it? These agents are now rarely used by any one; and it need scarcely be added, that nothing can be given as a solvent for concretions in the gall-ducts.

The lecturer stated, that there was never ground for uneasiness in cases of jaundice, unless when dependent on serious organic disease of the liver, which is usually accompanied by dropsy, or when produced by obstructions of the biliary ducts in those of broken down constitutions.

The lecture was concluded by a few observations on the

JAUNDICE OF THE INFANT, commonly known as the "yellow gum"—the term "gum" probably being a corruption of "gown," the infant seeming to be covered with the yellowness as with a "gown." It is not an uncommon occurrence in children soon after birth, and after continuing for a short time, it usually disappears of itself.

The child is generally drowsy and languid during its continuance, but the affection is rarely serious. It is very common in hot climates and in the warmer portions of this country.

The lecturer never saw a case which was of any importance. It seems to be dependent upon obstruction to the flow of bile, by a collection of viscid meconium or mucus, where the ductus communis choledochus opens into the duodenum.

The only treatment demanded is the administration of a gentle cathartic, as castor oil, or rhubarb and magnesia, to clear away the matters collected in the intestines.

